# Dilation of the Os Uteri and Perinæum during Labor

**Published:** 1868-12-15

**Authors:** George Kilner

**Affiliations:** Sullivan, Ill.


### DILATATION OF THE OS UTERI AND PERINÆUM DURING LABOR.

BY GEORGE KILNER, M.D., OF SULLIVAN, ILL.

*Read before the Esculapian Medical Society, Kansas, Ill.*

MEDICAL men who have had much experience in the practice of *Obstetrics*, must have been observant of the fact, that an unusual rigidity of the *os uteri* and *perinæum* are the principal causes of protracting the pain and agony of parturitions. The contractions of the *os* may come on every few minutes, and apparently with an amount of force sufficient for safety. Yet he is mortified to find, upon examination, the *os* undilated, and to all appearances undilatable, while the pains during this stage of labor we are speaking of, are often very distressing, and are denominated by women as a grinding or cutting sensation, frequently producing great anxiety, depression of spirits, and impatience to obtain relief, it now becomes a matter of importance to keep up the spirits, and maintain the confidence of the patient; this is easily done if the labor is a short one, but if it proceeds slowly through the night, and if the following day advances and still no prospect of a speedy termination, the patient begins to doubt whether she is receiving proper assistance, and those about her look suspiciously at you, they calculate the number of hours the labor has already lasted, they *wonder* it is not further advanced, and you are made to feel, both by looks and hints, (which are sufficiently intelligible), that your competency is thought to be rather questionable; when you find yourself in this situation, you will have considerable difficulty in persuading the females present, "*that all is right*," because they can not duly appreciate the obstacles to be overcome, in cases where the *os* remains unyielding for a protracted period. Now the question should arise in the mind of every humane accoucheur, What means may, with all confidence in their safety, be resorted to, to restore force to those natural processes? and thus remove all difficulties in the way to a speedy parturition. The usual routine of practice in such cases are well known — a *venesection*, a solution of *Ant et Pot Tart.* — *warm stupes to perinœum*, warm teas, *Ex. Belladonna*, *Opium*, *Ipecac*, *Chloroform*, etc., and then comes patience. But with what feelings of humanity can the attendant physician calmly advise patience to a woman well nigh exhausted by painful throes and agony, which, perhaps, may have already continued for twelve, eighteen, or twenty hours, or even more; when her frail and weak body, prostrate and helpless before him is agonized by fruitless contractile efforts of the uterus, to free itself of its burden, when with plaintive wails she beseeches him, at the end of every pain, to save her! who can sit listlessly by, under such circumstances, and advise *patience*, *be still* — she *can not* be still while racked by the dreadful exertions of the uterus. What then is the accoucheur to do? what can he do? simply resort to some measure to overcome the tension, or rigidity, of the parts implicated in delaying the advance of the fœtal head; but he has already tried bleeding, *Antimony*, warm teas, *Ex. Belladonna*, *Chloroform*, *Opium*, poultices, *Ipecacuanna*, etc., and yet hour after hour elapses, with little or no perceptible change under such circumstances should we be justifiable in using the knife, as recommended by some, for instance Dr. Marion Sims, who has won a world-wide reputation; see, also, Dr. Baker Brown's practice; also, Dr. Hilderbrandt, of Konigsburg, he gives a brief account of nine labors, in seven of which "primiparæ," advanced in life, suffered from rigidity of the os uteri, against which *Ipecac*, *Opium*, poultices, baths, bleedings, and *Chloroform*, were all unavailing. Incisions were made; after which all were fortunately terminated. Incisions were also made, with a like favorable result, in one case of convulsions, and one in "prolapsus of the cord." He remarks, "the operation is chiefly indicated, however, in morbid conditions of the vaginal portion of the cervix. Such as rigidity, hypertrophy, and malignant disease, for forced delivery, with a healthy cervix, the incisions should be six or eight in number, and not more than three lines in depth." Now, admitting that the womb and its appendages *will*, in many instances, bear such severe surgical treatment, when employed for surgical diseases, are we, therefore, to feel warranted in submitting any poor woman's womb, which should happen to be a little tardy in the completion of its high office of sending a human being into the world, and fulfilling one of its noblest physiological functions, to the tortures of surgical treatment.

As all these measures have been advised, in certain cases, by the highest obstetrical authorities, I would inquire how they will compare, in point of mildness, with the following treatment, when all other means fail, and before resorting to the knife or forceps, let the accoucheur, without fear or prejudice, try one other remedial agent, let him introduce a suppository, prepared after the following formula,

℞ Lobelin, grs. v.
Coca Butter, ʒj., into the rectum, immediately upon the subsidence of a pain, also, anoint the os with the same, by placing a small quantity upon the finger. Fifteen or twenty minutes retention is generally sufficient to produce a wonderful effect.

*Lobelin* is the most powerful relaxant in the Materia Medica, and one from which no danger need be apprehended, when used as above recommended. Its peculiar powers are speedily diffused by contiguous and continuous sympathy to the os, while the perinæum and supervening pains show a manifest dilatation of the os and perinæum, if hitherto rigid, yields readily to the advancing head. Never have I been more convinced of the superior efficacy of the above treatment, then in the attendance and delivery of cases during the past four years, as the following case will, among many others, show:

At 10 o'clock, P. M., March 29th, 1864, I was called to attend Mrs. T., primipara, American, aged twenty-six years, who had been in hard labor for sixteen hours previous to my being called in; upon my arrival, the midwife in attendance informed me that she supposed the head was too large to pass through the bones of the pelvis; she stated that the head had not advanced any for seven long hours previous to my arrival, and now, in her exhausted state, with a threatening of eclampsia, had sent for me to deliver the woman by the aid of instruments.

Upon an examination, I found no difficulty in the size of the pelvis, and the head was free and rotating against a rigid and firm perinæum, so all we would have to do were to use such means as would overcome the rigidity of the parts implicated, and produce a speedy relaxation to the parts indicated, for the poor woman was almost in a state of insensibility from the agonizing pain. The time for bleeding, etc., had passed, or to advise *patience*, but we simply introduced a suppository, prepared after the following formula,

℞ Lobelin, grs. v.
Coca Butter, ʒj., into the rectum, and anointed the fœtal head with the same, in a few minutes after the application the perinæum relaxed, apparently as thin as tissue paper, and the crown projected beneath the pubal arch, and, at the ensuing pain, the child was born. As to dilating the perinæum, this case satisfied me that the recent researches are correct, that this organ possesses contractive and dilative force, of a character not unlike that of the uterus. It is well known that its unyielding resistance often retards labor more then an unyielding os, especially in primiparous cases; indeed, an unyielding os and an unyielding perinæum are often concomitants.

On the 12th of June, 1866, about 1 o'clock, A. M., I received a call to attend Mrs. R., a thin, spare woman, in labor with her fourth child; the pains had been strong some hours before my arrival; upon an examination, I found an exceedingly rigid condition of the soft parts, and the os but slightly dilated. I put her upon the use of an antimonial solution, with *warm stupes*, to perinæum. I then lay down to await the effect of the medicine, with orders to call me whenever they should think it necessary. At six next morning I found my patient much exhausted, and in a continued agony of pain, and of fruitless pain, for even now I could with difficulty pass my index finger through the "ostium vaginæ," — but little dilation had ensued, and an unusual mass of rigid muscles seemed to line the pelvis and guard the outlet. Exceedingly discouraged, I administered *Chloroform*, in half drachm doses, mixed with sweet milk, every thirty minutes, until three doses had been taken; after waiting three hours, and no change having taken place, except for the *worse;* and amidst the fears of the husband, and cries of the woman for help, that she would die — the patient being a sensitive woman, and subject to hysterical fits. At this stage convulsions set in, and appeared to be of the usual character. Stertorous breathing, foaming at the mouth, stiffness of the extremities, beating of the cervical veins, teeth clenched, twitching of the facial muscles, mouth drawn on one side, etc., then followed a state of repose.

Under the impression that the woman would not survive the birth of the child, I concluded, as a last resort, before sending for counsel, to try the following,

℞ Lobelin, grs. v.
Coca Butter, ℨj.,

Mix into suppository. This I introduced into the rectum; also, anointed the os with the same, applying it with my finger, and lubricating the parts, in a short time I found, upon examination, that the os uteri had relapsed its rigid grasp upon the vertex of the child, and the whole crown was pressing against a soft, yielding perinæum, and without any more convulsions the child was born. I then put her upon the following,

℞ Chloroform, ℨj.

Gum Acacia, ℥j.

Aqua Camph, ℥j.,

Mix into an emulsion. Dose, teaspoonful whenever the pains require it. She made a safe recovery.

In conclusion I would say, *Lobelin* used thus in obstetrical practice, is a pure and safe relaxant; and in cases where the pains are irritable, os uteri rigid, perinæum unyielding, and secretions scanty, the above treatment will generally remove all difficulty — while the uterine contractions, and action of the abdominal muscles are not interfered with, and where bleeding may be objected to on account of a weakly or debilitated habit, at and after parturition, for the patient's safety, and in all cases of dry labor, he will find in such cases it will aid the flow of the proper secretions; it also obviates convulsions, when threatening in protracted labors, from causes cited above, by its excito-relaxant powers, changing the base of excitement from the brain to the rectum, and contiguous parts; and is also a sure remedy to facilitate labor, when retarded, and will bring the labor to a close, and save the time and suffering spent in long lingering cases, and assist nature to perform her function, with safety to both mother and child.

